# Effects of Biopsychosocial Interventions on Non-specific Chronic Low Back Pain and Its Related Disabilities among Students

**DOI:** 10.34172/jrhs.2022.103

**Published:** 2022-12-29

**Authors:** Rashid Heidarimoghadam, Younes Mohammadi, Ramin Kordi

**Affiliations:** ^1^Department of Ergonomics, School of Public Health, Hamadan University of Medical Sciences, Hamadan, Iran; ^2^Research Center for Health Sciences, Hamadan University of Medical Sciences, Hamadan, Iran; ^3^Sports Medicine Department, Faculty of Medicine, Sports Medicine Research Center, Institute of Neurosciences, Imam Khomeini Hospital Complex, Tehran University of Medical Sciences, Tehran, Iran; ^4^Department of Epidemiology, School of Public Health, Non-Communicable Disease Modeling Research Center, Hamadan University of Medical Sciences, Hamadan, Iran; ^5^Modeling of Noncommunicable Diseases Research Center, Hamadan University of Medical Sciences, Hamadan, Iran

**Keywords:** Biopsychosocial model, Low back pain, Musculoskeletal disease, Student health

## Abstract

**Background:** This study aimed to investigate the effects of biopsychosocial interventions on non-specific chronic low back pain (NSCLBP) and disabilities caused by it among Students.

**Study Design:** A two-group pretest-posttest randomized clinical trial.

**Methods:** The statistical population of the study was female students enrolled at the first-stage secondary school in Hamadan, Iran. A total of 200 students were selected through cluster sampling and randomized into two groups of intervention and control. The primary evaluation was performed by the Cornell Musculoskeletal Discomfort Questionnaire (CMDQ), the Health-Related Quality of Life (SF-36), the International Physical Activity Questionnaire-Short Form (IPAQ-S), the World Health Organization Disability Assessment Schedule (WHODAS), and the visual analogue scale (VAS). Upon developing and implementing the biopsychosocial model-based interventions for ten weekly two-hour sessions, the secondary evaluation was fulfilled, and the extracted data were analyzed using the IBM SPSS version 21.

**Results:** The independent-group t-test results revealed that the mean scores of quality of life (QOL) and physical activity significantly elevated in the intervention group, compared to the control. In addition, the mean value of disabilities, the amount of disorder in the lumbar region, and the VAS scores in the intervention group substantially declined compared to the control group.

**Conclusion:** The significant variations in the biopsychosocial factors demonstrated that the development of some interventions based on the bio-psychosocial model (BPSM) could help manage the NSCLBP and its ensuing disabilities. Therefore, the BPSM-based interventions could be exploited to minimize musculoskeletal disorders (MSDs) in students.

## Background

 Prevalence rate of the low back pain (LBP) among children and adolescents, was increases and its prevalence in has reached the level of prevalence in adults.^1^ Therefore, the LBP has turned into one of the causes of disabilities across the world and even a common and costly condition affecting public health.^2,3^

 Non-specific chronic low back pain (NSCLBP) that defined as pain, muscle tension, or stiffness localized below the costal margins (ribs) and above the inferior gluteal folds with or without leg pain.^4^ The incidence, prevalence, and disability-adjusted life year of LBP are to build up 1.4-fold by 2050.^5^ The LBP incidence rate in this age group is 7-74%.^6,7^ Accordingly, this health condition seems to affect their quality of life (QOL) and may even diminish their ability to practice daily activities.^8^ Notably, the loads carried by students can significantly induce musculoskeletal pains in this age group, and school bags may be specifically the main factor leading to musculoskeletal disorders (MSDs) among them.^9^

 In this line, numerous studies have reflected the relationship between carrying school bags and musculoskeletal pains. The maximum of 10-15% of the body weight has recently been advocated as the loads carried by students.^10^ As per the Global Burden of Disease Study released by the World Health Organization (WHO), neck pain and other MSDs have been thus far ranked in the fourth and tenth places for the age group of 15-19, respectively.^11^

 At COVID-19, most countries swapped their courses with online ones, wherein students would spend hours on electronic devices.^12^ The use of such gadgets could be associated with musculoskeletal complaints among them, as a major concern due to its negative impacts on the ability to perform daily activities.^13^ Research shows that children and adolescents with LBP require more health care services. They are more likely to have high levels of absenteeism and much disruption in their school and physical activities.^14-16^ In addition, the growing body of evidence implies that the onset time of the LBP is between the ages of 10 and 14, escalating with age and approaching adulthood around 18. Schools are endowed with many potentials to aid students develop the related knowledge and skills to be more healthy.^17^ A recent study in Spain reported the lifetime prevalence of LBP by 44.5% in a cohort of 1500 adolescents from the Valencian Community aged 12-18.^16^ The prevalence rate in girls (50.3%) was also higher than that in boys (38.9%) and reached 36.9% at the age of 13.^17^

 With the current breakthroughs in technology trends, smartphones have earned more common use and popularity around the world.^18^ The postures taken by individuals while using smartphones have been linked to the physical pains induced by using smartphones for texting purposes.^19^

 The NSCLBP is typically characterized by psychosocial and biophysical factors, comorbidities, as well as pain processing mechanisms.^20^ Accordingly, the bio-psychosocial model (BPSM), considering the BPSM factors and their interactions, seems more effective in providing information to promote health and prevent many diseases.^21^ Such factors that have often been associated with the risk of NSCLBP include age, being female, low level of education, high body mass index (BMI), sedentary lifestyle, smoking habits, anxiety, depression, fear-avoidance beliefs, maladaptive illness perceptions, and inadequate social support.^20^ Even if some studies have so far utilized combined psychological-biological consequences, those reflecting on the psychosocial effects among students have been yet limited. In previous studies, the consequences of pain have been less discussed. The completed studies have mostly focused on the physiological pain itself, and the effect of psychosocial interventions on outcomes such as the inability to engage in social activities and perform physical activities is unclear. Furthermore, effectiveness of virtual education, in areas such as the subject of the present study is still unclear. Moreover, according to the socio-psychological situation, the difference between girls and boys, our knowledge in the mentioned field is limited, and the results of this study can be helpful in this field. Against this background, the present study aimed to shed light on the factors affecting the prevalence rate of MSDs in students, using the BPSM, and then design some interventions to improve the level of pain, disability, and QOL in this age group.

## Materials and Methods

###  Participants

 The present study was a two-group pretest-posttest randomized clinical trial on the statistical population of female students enrolled at the first-stage secondary school in Hamadan, Iran. In accordance with the similar studies,^22^ the sample size was determined as 200 individuals (i.e., 100 students in the intervention group and 100 controls). To begin the study, the first-stage secondary schools for girls in Hamadan were divided into four regions based on their geographical, cultural, and economic characteristics. Afterward, the number of schools in each region was ascertained, and then a number of schools were selected as the clusters in respect of the required sample size. Of note, four first-stage secondary schools were randomly selected in each region as much as possible, and each one was randomized into the intervention or control groups to prevent information exchange. The cluster sampling also continued until the desired number of samples was reached. The inclusion criteria were the students’ willingness to contribute to the study, having spinal pains, and obtaining parental consent. On the other hand, the exclusion criteria at the intervention stage were having a history of underlying chronic diseases (e.g., hypertension, diabetes mellitus, and heart disease), suffering from congenital defects, having defects from secondary causes (e.g., accidents), not attending more than three intervention sessions, and experiencing physical problems or illnesses during the study.

###  Data collection instruments

 The Cornell Musculoskeletal Discomfort Questionnaire (CMDQ), the 12-item Short Form Survey (SF-12), the International Physical Activity Questionnaire-Short Form (IPAQ-S), the World Health Organization Disability Assessment Schedule (WHODAS), and the visual analogue scale (VAS) were the main data collection instruments employed in this study. Age, gender, weight, height, LBP history, and level of physical activity were among the demographic variables investigated here.

###  Cornell Musculoskeletal Discomfort Questionnaire 

 As one of the well-known data collection instruments developed by Hedge, Morimoto, and McCrobie in 1999 to evaluate the MSDs, the CMDQ has been prepared at three stages, viz., level of discomfort, intensity rate, and interference, with a map to analyze 12 body parts, and 20 in total.^23^ Repetition score (never = 0, 1 to 2 times a week = 1.5, 3 to 4 times a week = 3.5, everyday = 5 and several times a day = 10), discomfort score (3, 2, and 1) and Interference with work (3, 2, and 1), are multiplied together, and the total score is equal. The CMDQ is currently applied in the United States and other countries, and its validity and reliability have been already checked and confirmed in Iran.^24^

###  Health-Related Quality of Life 

 The 36-Item Short Form Survey (SF-36) contained 36-items under eight subscales to measure QOL in two general dimensions, namely, physical and mental health. The first four subscales include physical functioning, role-physical, bodily pain, and general health within the physical health dimension, and the last four subscales, representing the mental health dimension, are vitality, social functioning, role-emotional, and mental health. All items are scored on a Likert Scale; however, the scoring range of the items is different. Confirming the validity and reliability of the SF-12 in Iran, Montazeri et al normalized and retranslated it into Farsi and then applied it to 4,163 individuals aged 15 and above (i.e., the mean age of 35.1), including women (52%). In the present study, only the physical health dimension was taken into account.^25^

###  International Physical Activity Questionnaire-Short Form 

 The IPAQ-S items are related to performance (i.e., physical activity over the last week). According to this questionnaire, the pattern and intensity of the physical activities practiced in the past seven days can be determined. In this way, the activities such as aerobics, high-speed cycling, mountain climbing, and basketball, which require over six calories per minute, are called intense physical activities. Other activities, such as volleyball, badminton, and cleaning the room that demand 3-6 calories per minute, are considered moderate cases. In addition, activities lasting below 10 minutes are excluded. The total energy intensity (TEI) of all physical activities performed over the last week was also calculated in line with the IPAQ guidelines. Thus, the TEI value, ranged between 0 and 599 Week/Cal/Met, meant poor physical activity; that of 600-3000 Week/Cal/Met, denoted moderate physical activity; and the one above 3000 Week/Cal/Met, represented intense physical activit.^26^ The Iranian version of this questionnaire had been further reviewed by Bakhtari Aghdam et al, indicating an acceptable content validity, viz., the content validity index (CVI) of 0.85 and the content validity ratio (CVR) of 0.77. Considering its Cronbach’s alpha coefficient, the internal consistency of the IPAQ-S was reported to be equal to 0.7.^27^

###  World Health Organization Disability Assessment Schedule 

 The 12-item WHODAS is utilized to measure behavioral disability in the last 30 days, scored based on the degree of difficulty in performing the desired activity from 0 (i.e., no problem) to 4 (viz., inability to perform the activity). The total score is obtained from the sum of all items. The WHODAS was designed to measure efficiency and disability in keeping with the comparison of International Classification of Diseases (ICD) in different societies. Its conceptual framework is based on the International Classification of Functioning, Disability, and Health (ICF), approved by 191 countries as the standard system for classifying health status in people. The ICF also belongs to the classifications released by the WHO, and is directly related to the ICD_10. The validity and reliability of the WHODAS had been previously established in domestic and international studies.^28^

###  Visual analogue scale 

 The VAS, as a 10-cm vertical line numbered from 0 to 10, is employed to measure the levels of pain. In this scale, 0 and 10 indicate the highest intensity of pain and the absence of pain, respectively. According to this data collection instrument, the participant was asked to determine the intensity of one’s pain over the last week using a pencil.^29^ The validity, reliability, and sensitivity of the VAS in acute and chronic diseases and cancer pain had been previously confirmed in various studies.^30^ The validity of this scale was thus estimated at 0.76-0.86, and its reliability was equal to 0.70-0.60.^31^ In addition to its high reliability and validity, the reason for implementing this scale was the ease of its completion.

###  Statistical analysis

 Upon completing the data collection instruments, the data were imported into the IBM-SPSS Statistics software package (version 21) for further analysis. A *P* value < 0.05 was considered statistically significant. Moreover, descriptive statistics, such as frequency distribution and percentage, and the indices of central tendency and dispersion were utilized to describe the data. The independent-group t-test was ultimately employed to test the research hypotheses.

###  Interventions

####  Control group

 The control group received no intervention; however, the BPSM-based interventions were performed on the control group members after the study was completed to observe the ethical issues.

####  Intervention group

 The students, teachers, and parents in the intervention group were provided with physical, mental, and social education during online and face-to-face sessions. Some educational sessions were also designed in the form of videos. In total, ten two-hour sessions were completed for ten weeks, and a total of 2000 person-hours of training were conducted. The educational videos contained various topics, from the introduction of ergonomics to students to the application of ergonomics in life and daily interactions. Moreover, the online sessions were specially designed before their implementation, and their electronic content was developed. Of note, the educational sessions were lecture-based, reflecting on the knowledge of ergonomics, spine anatomy, MSDs, the LBP, along with the risk factors of such conditions among students. Additionally, questions and answers were raised regarding ergonomics in school settings for students, and practical education was provided through the educational videos on spine and back problems.

 The students were further encouraged by an ergonomics specialist to correct and maintain appropriate postures for sitting, studying, sleeping, and even working with smartphones, tablets, laptops, and other educational devices. The primary evaluation was done via questionnaires, and then the students’ spine, knees, and soles were examined by a specialist in sports physiology to identify possible abnormalities. In line with the diagnosis of the complications, the problems such as the LBP, flatfoot, scoliosis, rounded shoulders, hunchback, and club foot were detected among the students. To intervene, the students were taught some short-term stretching exercises related to the back and neck area for 10-12 minutes after every 45 minutes at home, using the home exercise protocols, along with putting much emphasis on the importance of maintaining spine health and the problems caused by the lack of health in these areas. These exercises were carried out for six months under the direct supervision of an ergonomics specialist. Notably, the students diagnosed with acute conditions in their spine were referred to the healthcare facilities for further treatment. The exercises were provided to the students through the SHAD application (SHAD is an application created with the aim of providing virtual education to students). Moreover, a booklet containing information about the LBP and the ways to prevent it was provided to the students electronically. An expert and a psychologist were recruited to perform the psychological and social interventions and teach the educational sessions, whose content was to familiarize the students with acute and chronic stress and ways to control, manage, and deal with it. During these sessions, the students were fully given the opportunity to address the factors affecting stress, and then they were taught the practical and specialized solutions to tackle it. Table 1 outlines the BPSM-based interventions implemented in this study.

**Table 1:** Interventions based on the biopsychosocial model

**Interventions**
Physical
1. Designing and teaching corrective movements related to back pain, the low back pain (LBP), scoliosis, neck pain, shoulder pain, flatfoot, and club foot 2. Teaching how to properly carry a school backpack, choose an ergonomic and suitable one, adopt proper postures while studying, and use smart devices correctly and ergonomically, such as smartphones, tablets, laptops, and desktop computers 3. Teaching how to sleep correctly, and choose the right pillow with ergonomic features 4. Teaching how to improve the ergonomics of the desktop computer station at home 5. Teaching how to lift and carry loads and do repetitive movements 6. Teaching about weight control and the effect of BMI on the LBP
Psychological
1. Teaching about stress and anxiety management and control skills 2. Teaching about the impact of psychological factors, such as stress, anxiety, and depression on the LBP and the psychosomatic pain in students 3. Teaching about the concept of mental health, factors affecting mental health, and how beliefs, moods, and knowledge could affect the stress perceived by the surrounding environment
Social
1. Teaching how to establish correct communication in families and communities 2. Teaching parents and teachers about the LBP, corrective movements, and healthy lifestyle 3. Teaching families about the life-work balance and the importance of their role in raising children 4. Teaching about the effects of smart devices on intra-family communication and their impact on isolation, depression, and avoidance in children's society

## Results

 Descriptive results are presented in Table 2. The mean age of the participants is 14.2 years, and mean height and weight is 15,803 and 54.8, respectively. Accordingly, mean score for Physical health, Disability score, Backache, Physical activity, and Vas is 83.6, 7.2, 13.2, 2.5, and 2.5, respectively.

**Table 2:** Descriptive presentation of the participants of the study

**Variables**	**Mean**	**SD**	**Min**	**Max**
Age	14.2	0.96	12	16
Height	158.3	23.1	135	175
Weight	54.8	13.9	25	88
Physical health	249.5	83.6	95	400
Disability score	7.9	7.2	0	30
Backache	6.6	13.2	0	90
Physical activity	3.4	2.5	0	10
Visual analogue scale	3.4	2.5	0	10

 The demographic characteristics of the sample and the investigated variables in the two groups were compared before the intervention. The results are presented in Table 3.

**Table 3:** Demographic and baseline characteristics of the participants

**Variables**	**Range**	**Intervention group**		**Control Group**		**Test statistic value**	***P *** **value**
		**Mean**	**SD**	**Mean**	**SD**		
Age (y)	12-16	14.30	0.94	14.20	0.95	t = 2.17	0.100
Body mass index (kg/m^2^)	12.19-60.22	21.52	6.10	20.59	3.81	t = 0.09	0.220
Average course grades	12-20	18.05	1.74	18.3	1.78	t = 0.97	0.330
Physical health	30-400	256.70	84.05	242.00	82.90	t = 1.20	0.230
Disability score	0-30	7.40	7.50	8.40	7.00	t = 1.04	0.300
Backache score	5-90	6.90	13.90	6.10	12.40	t = 0.49	0.680
Visual analog scale	2-10	3.30	2.30	3.40	2.80	t = 0.24	0.710
Physical activity	1-3	1.50	0.89	1.40	0.63	t = 0.56	0.140

Table 3 demonstrates no significant difference between intervention and control groups in terms of age, BMI, average course grades, and baseline scores of physical health, disability, backache, visual analog scale, and physical activity (*P* > 0.05).

Table 4 presents the scores of physical health, disability, back pain, visual analog scale, and physical activity for two study groups. Accordingly, the physical health score in the intervention group (292.1 ± 67.7) is higher than the control group (243.8 ± 84.8). The *P* value of the test indicates that this difference is statistically significant (*P* < 0.001). In terms of disability score, the difference between the intervention group and control group is significant (*P* = 0.02). The mean score for intervention groups is 6.4 with a standard deviation of 5.8 and for the control group is 8.8 with a standard deviation of 7.4. The result of the backache score after the intervention phase indicates a significant difference between the groups of the studies. The score in the intervention group is higher than the control group by 3.1 (*P* = 0.04). The score of VAS in the control group was significantly higher than in the intervention group (*P* < 0.001). Table 4 demonstrates that the mean VAS for the control and intervention groups is 3.3 and 1.8, respectively. In terms of physical activity, we found that score of this dimension in the intervention group was higher than the control group. The mean score for the intervention and control groups was 1.73 was 1.4, respectively (*P* < 0.001).

**Table 4:** Post-test scores of the intervention and control groups

**Variables**	**Intervention Group**		**Control Group**		***P *** **value**
	**Mean**	**SD**	**Mean**	**SD**	
Physical health	292.1	67.7	243.8	84.8	0.001
Disability	6.4	5.8	8.8	7.4	0.020
Back pain	3.8	7.7	6.9	13.3	0.040
Visual analog scale	1.8	1.7	3.3	2.6	0.001
Physical activity	1.7	0.6	1.4	0.6	0.001

## Discussion

 The study results revealed that the NSCLBP intensity and the resulting disabilities significantly decreased after the BPSM-based interventions completion. This suggested that exercise and ergonomic recommendations could have significantly relieve pain and minimize disabilities caused by NSCLBP. The findings reported in Bandpei -Bandpei^32^ investigating the impact of exercise and ergonomic recommendations on the treatment of prenatal LBP had further established that regular pre/postnatal exercise and compliance with the ergonomic principles could significantly reduce pain and disabilities induced by the LBP at this time. The results of a study by Jafari-Nodoushan^33^ also confirmed that the amount of pain and discomfort in nine body areas in students had significantly amplified during the COVID-19 pandemic compared to that before it. Moreover, The effect of using ergonomic interventions to reduce MSDs has been shown in various studies.^34,35^

 The prevalence rate of the NSCLBP in the control group was 13.3 ± 6.9, and this value significantly declined to 7.7 ± 3.8 after the BPSM-based interventions in the present study (*P* < 0.04). This was attributed to the educational biopsychosocial program. This was consistent with the result in Ekpenyong et al,^36^ investigating the MSDs, stress, and home environment factors among students in Malaysia. They further reported a significant relationship between MSDs in 20 body parts (*P* < 0.05) and physical activity, interpersonal relationships, academic, and environmental stress. Additionally, the occurrence of the MSDs in seven body parts had shown a significant difference (*P* < 0.05), as compared with those arising from the home environment factors. In conclusion, the students had experienced a high prevalence rate of MSDs, associated with stress and the home environment. In the interventions designed based on the biopsychosocial model, there was training in corrective movements in the biological part, which strengthened the muscles and, as a result, reduced the pain. Moreover, stress management training has helped to alleviate psychosomatic pain.

 In the present study, the students’ physical activities for six months established that the mean score of this dimension was higher in the intervention group (1.73) than in the controls (1.4) (*P* < 0.001). According to Khodabakhshi et al,^37^ analyzing the effect of an eight-week corrective exercise intervention program on computer users, the upper and lower extremity pains had significantly moderated by 38.3% (*P* = 0.001) and 38.7% (*P* = 0.006), respectively. Furthermore, Karimian et al,^38^ investigating the nurses of Al-Zahra hospital, found that the MSDs in the lower back had been higher than in other areas, and such conditions had significantly dropped (about 50%) after eight weeks of corrective movements and ergonomic interventions. Researching physical activity, sedentary behavior, and sleeping habits in Italian students during the COVID-19 quarantine, Luciano et al^39^ had also shown a downward trend in physical activity and an increase in sitting and sleeping time before and during the quarantine period in the sixth-grade students (*P* < 0.01).

 Here, the mean score of the physical health in the intervention group (292.1 ± 67.7) was higher than that in the controls (243.8 ± 84.8). The variations in the students’ QOL also significantly reduced the LBP. Accordingly, the disabilities were not just physical, but the NSCLBP in different types and intensities had affected some aspects of their mental and social life; therefore, paying attention to the mental and social aspects of disability and using psychological interventions to reduce the disabilities could be effective. Heydari Moghadam et al,^40^ in their study on the patients with the LBP had similarly reported a significant positive relationship between pain intensities and the socialization subscale. Furthermore, a significant positive relationship was found between pain intensity and social connection, life activity, presence in society, and general disability. In this regard, the regression analysis outcomes by Mitchell et al^41^ and Ben Ayed et al^42^ on the psychosocial factors affecting the LBP in female students had also revealed that stress, coping styles, physical activities, spine kinematics, and age had all independently contributed to the LBP, accounting for 23% of the variance. The reports in Sadeghi Yarandi et al^43^ on the relationship between individual, physical, and psychosocial risk factors and the MSDs and their resulting disabilities in airport security personnel had further established that lifestyle parameters, stress, and mental workload had been among the most important risk factors for the prevalence of the MSDs and work-related disabilities in these employees. Therefore, corrective measures seemed essential since they could control individual, physical, and psychosocial risk factors.

 One of the limitations of the present study is that it was conducted on female students, and the biopsychosocial factors and their consequences may be different in male students. Furthermore, caution should be taken in generalizing the results of this study.

## Conclusion

 The results of this study showed that the interventions designed based on the biopsychosocial model, in addition to reducing perceived pain in students with NSCLBP, can also improve their ability and QOL. Therefore, adopting a multifaceted approach in interventions related to NSCLBP in students can be effective.

**Highlights**

- The biopsychosocial model can effectively reduce perceived pain of non-specific LBP.
- The biopsychosocial model can effectively improve the capabilities of people with non-specific LBP.

## Acknowledgments

 The authors would like to express their gratitude to the esteemed principal of the first-stage secondary school for girls in the city of Hamadan, Iran, and the respectable parents and loved students for their contribution to this research project.

## Conflict of interest

 The authors declare no conflict of interest.

## Ethical approval

 This study was registered in Iranian Registry of Clinical Trials with registration number IRCT20220220054076N1.

## Funding

 Vice Chancellor for Research and Technology of Hamadan University of Medical Sciences supported this research (Grant number: 140107125654).
